# Reference loci for RT-qPCR analysis of differentiating human embryonic stem cells

**DOI:** 10.1186/1471-2199-14-21

**Published:** 2013-09-12

**Authors:** Liesbeth Vossaert, Thomas O’Leary, Christophe Van Neste, Björn Heindryckx, Jo Vandesompele, Petra De Sutter, Dieter Deforce

**Affiliations:** 1 Laboratory for Pharmaceutical Biotechnology, Ghent University, Harelbekestraat 72, Ghent 9000, Belgium; 2 Department for Reproductive Medicine, Ghent University Hospital, De Pintelaan 185, Ghent 9000, Belgium; 3 Center for Medical Genetics, Ghent University Hospital, De Pintelaan 185, Ghent 9000, Belgium

**Keywords:** Reverse transcription quantitative PCR, Normalization, Reference genes, Alu repeats, Human embryonic stem cells, Stem cell differentiation

## Abstract

**Background:**

Selecting stably expressed reference genes is essential for proper reverse transcription quantitative polymerase chain reaction gene expression analysis. However, this choice is not always straightforward. In the case of differentiating human embryonic stem (hES) cells, differentiation itself introduces changes whereby reference gene stability may be influenced.

**Results:**

In this study, we evaluated the stability of various references during retinoic acid-induced (2 microM) differentiation of hES cells. Out of 12 candidate references, *beta-2-microglobulin*, *ribosomal protein L13A* and Alu repeats are found to be the most stable for this experimental set-up.

**Conclusions:**

Our results show that some of the commonly used reference genes are actually not amongst the most stable loci during hES cell differentiation promoted by retinoic acid. Moreover, a novel normalization strategy based on expressed Alu repeats is validated for use in hES cell experiments.

## Background

Human embryonic stem (hES) cells, derived from the inner cell mass of a blastocyst stage embryo, are able to differentiate into all cell types of the adult body and have the potential for unlimited growth [1-4]. As a consequence of their pluripotency and self-renewal capacity, hES cells are ideal for investigating the basic mechanisms of development and cell differentiation. In addition, they may be a source of differentiated cells of a particular cell type, to be used in toxicity screening, cell replacement therapies and many other applications [3,5].

Pluripotent cells are characterized by several features, such as the expression of pluripotency factors (Oct4 (encoded by *POU5F1*), Nanog (*NANOG*) and Sox2 (*SOX2*)), the presence of specific cell surface antigens (e.g. SSEA-3, SSEA-4, TRA-1-60 and TRA-1-81), and distinct chromatin signatures [3,6-9].

To date, the molecular basic mechanisms of (spontaneous) hES cell differentiation and development are largely unknown [4,10]. Differentiation can be induced in vitro under specific culture conditions, such as the addition of retinoic acid, a morphogen commonly used for multilineage differentiation of ES cells in general and for specific development along the neural lineage [11-13]. Amongst other techniques, reverse transcription quantitative polymerase chain reaction (RT-qPCR) is very well suited for monitoring pluripotency and differentiation, as it allows accurate messenger RNA quantification of numerous samples at the same time [14,15]. In the context of hES cell characterization, RT-qPCR is applied for evaluating the expression of the transcription factors Oct4 and Nanog, since the expression of these core pluripotency circuitry members [9] decreases significantly within a few days after onset of differentiation [10,11].

For proper RT-qPCR data evaluation, several variables need to be taken into account. These include sample handling and storage, starting material quantity and quality, efficiency of different enzymatic reaction steps and overall transcriptional activity differences between cells [14,16].

To correct for these variables, different normalization methods have been reported. Gene expression levels can be standardized to cell number, however, it is not always possible to obtain an accurate enumeration of cells. In addition, this strategy does not consider possible insufficient enzymatic reaction efficiencies [14,17]. Alternatively, data are normalized for RNA mass quantity, although this is not always representative for the mRNA content. Ribosomal RNA molecules make up the major part of the total RNA mass and may be regulated, thus resulting in a variable rRNA/mRNA ratio [14,16-18]. The most frequently utilized strategy is the inclusion of one or preferably more reference genes as an internal standard. The expression of these references should ideally not vary between cells of interest or as a consequence of experimental handling [14,16,17,19].

Selecting stable reference genes is critical for correct interpretation of RT-qPCR data. However, when studying differentiating hES cells, proper reference gene selection is not straightforward. Differentiation does not only include various morphological changes, but also major alterations in gene expression levels of numerous genes. The regulation of some reference genes may be associated with these cellular changes, hence the stability of the used references has to be evaluated. The available differentiation protocols may induce distinct gene expression variability, which impedes finding stably expressed reference genes over the different samples and making protocol-dependent optimization required [10,14,16,17,19,20].

In this study, we emphasize the importance of determining suitable reference genes by performing an expression stability analysis for retinoic acid induced differentiating hES cells, using the geNorm^PLUS^ algorithm in the qbase^PLUS^ software (Biogazelle) [14,21]. The possibility of co-regulation was reduced by opting for 11 candidate reference genes from different functional categories [14]. In addition to these candidates, a new normalization strategy was applied, based on the measurement of expressed Alu repeats.

Alu insertions are repetitive DNA sequences, approximately 300 base pairs (bp) long and occurring generally at high copy number in introns, 3′ untranslated regions (UTR) of genes and intergenic genomic regions [22]. These short interspersed mobile elements are not equally spread throughout the human genome, since they preferentially accumulate in gene-rich regions [16,22]. In total, Alu elements comprise more than 10% of the genome mass, thus being the most abundant of all mobile elements and they are divided in several well-conserved subfamilies (e.g. AluSq, AluSx, AluY) [16,22,23]. Alu repeats, named after a recognition site for the restriction enzyme Alu I, are thought to be amplified by retrotransposition, a process in which the Alu element is transcribed by RNA polymerase III, followed by reverse transcription and incorporation into the genome [16,22-24].

Because of their genome-wide distribution, including in the 3′ UTR of protein coding genes, individual gene expression variability in the cells of interest will not substantially influence total Alu element expression. This feature makes the Alu repeats a valuable and interesting strategy for RT-qPCR normalization for biological systems such as differentiating stem cells [16,22].

## Results

**Figure 1:**
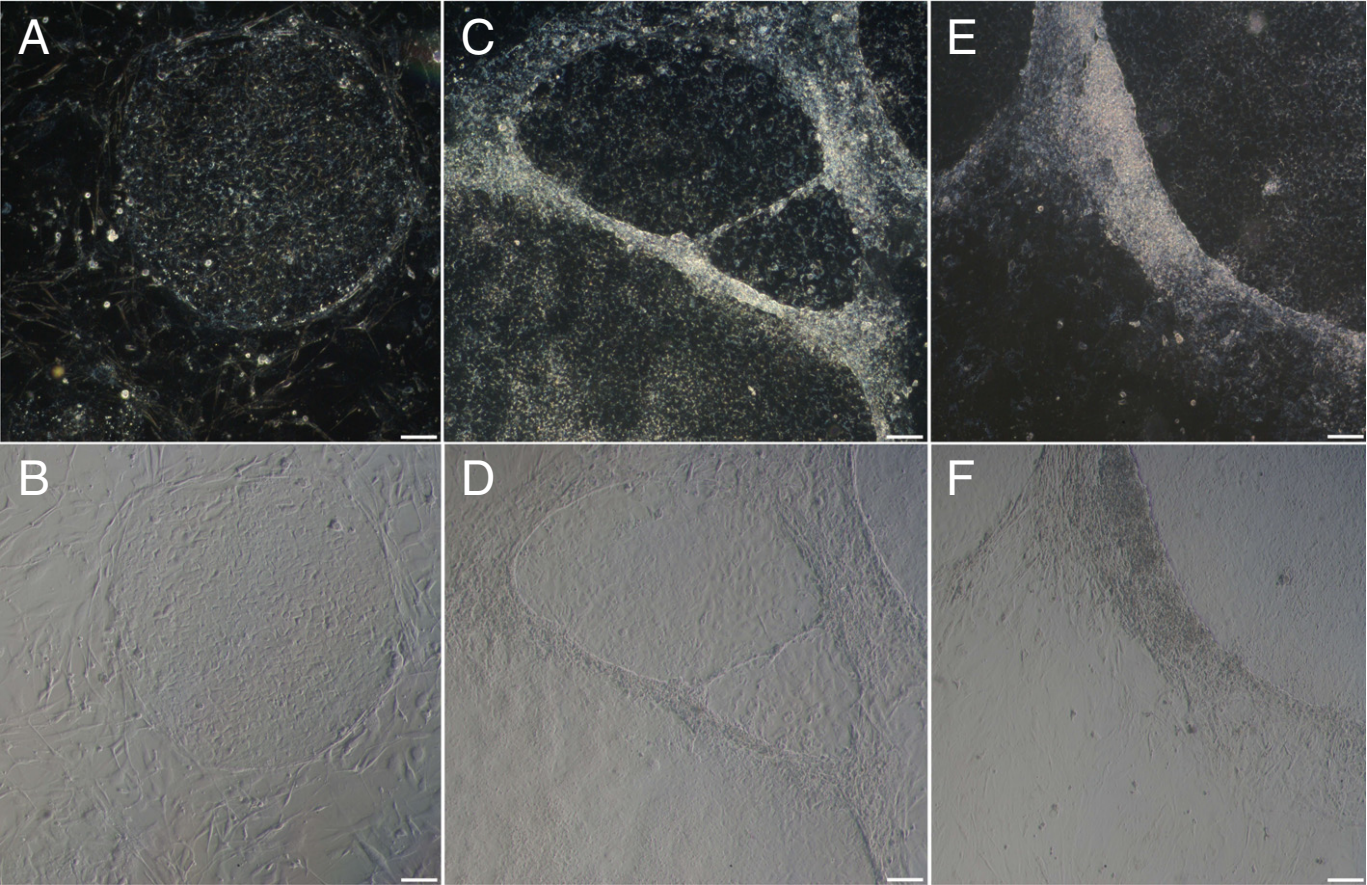
**Morphological evaluation of human embryonic stem cell culture.** Comparison of an undifferentiated colony **(A**: phase contrast, **B**: bright field image**)** with differentiated hES cells on day 3 **(C**, **D)** and day 5 **(E**, **F)** after retinoic acid induction (scale bars = 100 μm).

Human ES cells were induced to differentiate for several days by addition of retinoic acid to the culture medium. The fading undifferentiated state of hES cells was assessed morphologically, using light microscopy. Differentiation was initially visible at the colony periphery where cells start to pile-up, and in comparison to undifferentiated cells, differentiating colonies lost their round shape with well-defined borders, as illustrated in Figure 1.

To confirm differentiation, the expression levels of pluripotency genes *POU5F1* and *NANOG* can be followed over time using RT-qPCR. For this purpose, the most stable normalization references were determined by means of a stability analysis using the geNorm^PLUS^ application in qbase^PLUS^. The stability of 12 candidate references (see Table 1 for gene names and function) was analyzed for a first series of samples, which were isolated every 24 hours during 8 days of differentiation (= Experiment 1). In the stability ranking, *β-2-microglobulin* (*B2M*), *ribosomal protein L13A* (*RPL13A*) and Alu repeats (AluSq) were found to be the most stable reference loci for this experimental set-up (reference stability ranking displayed in Table 2). The stability measure (M value, see Additional file 1) of these three references varied from 0.277 to 0.290, which indicates a high stability in comparison to the other candidates, as M values up to 0.500 are considered acceptably stable for homogenous samples [21]. In the determination of the optimal reference number, two appeared to be sufficient for RT-qPCR normalization, as the pairwise variation (V value) was approximately 0.09 for upgrading from two to three reference loci (V2/3). V values less than 0.15 indicate that increasing the number of references would not add any more significant value to the assay.

**Table 1:** Reference loci and target genes included in the experiments

**Reference**	**Name**	**NCBI RefSeq**	**Function**	**Amplicon size (bp)**	**Exon location**
ACTB^*^	Beta-Actin	NM_001101	Cytoskeletal protein	173	7p22
B2M^§^	Beta-2-microglobulin	NM_004048	Major histocompatibility complex I component	86	15q21-q22
GAPDH^*^	Glyceraldehyde-3-phosphate dehydrogenase	NM_002046	Oxidoreductase in glycolysis and gluconeogenesis	111	12p13
HMBS^*^	Hydroxymethyl-bilane synthase	NM_000190	Porphyrin metabolism and heme synthesis	64	11q23
HPRT1^*^	Hypoxanthine phosphoribosyl-transferase 1	NM_000194	Purine synthesis in salvage pathway	94	Xq26
PPIA^*^	Peptidylprolyl isomerase A (Cyclophilin A)	NM_021130	Catalyzation of cis-trans isomerization of proline imidic peptide bonds	71	7p13
RPL13A^*^	Ribosomal protein L13A	NM_012423	60S ribosomal subunit structural component	126	19q13
SDHA^*^	Succinate dehydrogenase complex, subunit A	NM_004168	Electron transporter in citric acid cycle and respiratory chain	86	5p15
TBP^*^	TATA box binding protein	NM_003194	General transcription factor for RNA polymerase II	89	6q27
UBC^*^	Ubiquitin C	NM_021009	Involved in protein degradation	133	12q24
YWHAZ^§^	Tyrosine 3-monooxygenase / tryptophan 5-monooxygenase activation protein, zeta polypeptide	NM_003406	Signal transduction; binds to phosphorylated serine residues on several signaling molecules	94	2p25
AluSq	Alu repeats, subfamily Sq				
POU5F1^§^	POU class 5 homeobox 1, Oct4	NM_002701.4	Marker for embryonic stem cell pluripotency	130	6p21
NANOG^*^	Nanog homeobox	NM_024865.2	Marker for embryonic stem cell pluripotency	109	12p13

^*^exon-spanning primer pair; ^§^forward and reverse primer localized in the same exon.

**Table 2:** Reference stability analysis

	**Experiment 1**	**Experiment 2**	**Experiment 3**
1	B2M	B2M	B2M
2	AluSq	AluSq	RPL13A
3	RPL13A	RPL13A	AluSq
4	HPRT1	GAPDH	PPIA
5	YWHAZ	SDHA	GAPDH
6	TBP	TBP	HPRT1
7	HMBS	YWHAZ	TBP
8	ACTB	ACTB	YWHAZ
9	GAPDH	HPRT1	HMBS
10	PPIA	PPIA	SDHA
11	SDHA	HMBS	ACTB
12	UBC	UBC	UBC

Reference stability was analyzed for three independent hES cell differentiation experiments, applying the geNorm^PLUS^ algorithm. Results are displayed with decreasing stability from top to bottom (specific M values can be found in Additional file 1).

As a confirmation for this first experiment, a reference stability analysis was performed during two more hES cell differentiation experiments. For experiment 2 samples were collected every 24 hours during 6 days, and a third series was collected every 4 hours during day 3, 4 and 5 after onset of differentiation (= Experiment 3). Again, the same three reference loci were found to be the most stable in both experiments (Table 2). The M values for *B2M*, *RPL13*A and AluSq varied from 0.203 to 0.221, and from 0.378 to 0.386 for Experiment 2 and 3 respectively (all M values can be found in Additional file 1). Also in both cases, two references were shown to be enough for normalization, considering the low V2/3 values.

Subsequently, the expression data of *POU5F1* and *NANOG* were normalized applying two different reference sets: relative quantification using three commonly used genes (*GAPDH*, *ACTB* and *PPIA*) versus the three most stable references determined in the analyses described above (*B2M*, *RPL13A* and AluSq).

**Figure 2:**
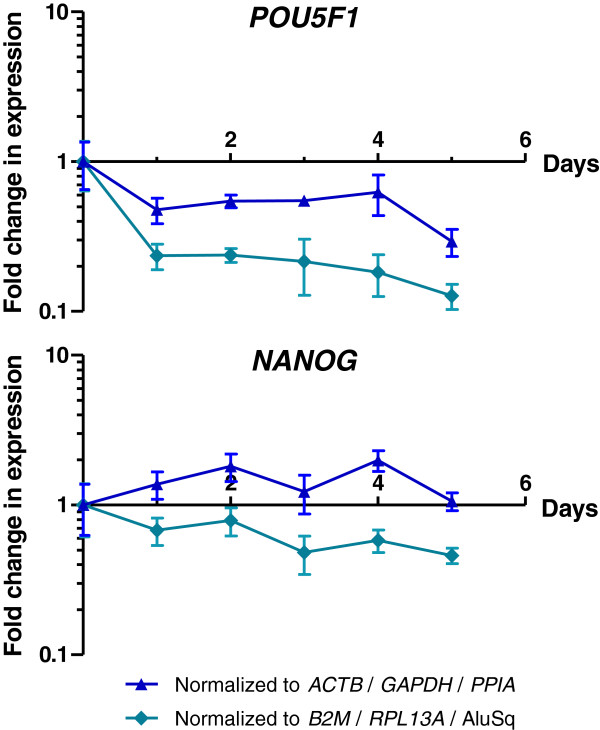
**Relative quantification of *POU5F1 *and *NANOG.*** The importance of proper reference gene selection is illustrated by comparing normalized data of the pluripotency factors Oct4 and Nanog to different reference sets. The Cq values for the pluripotency genes *POU5F1* and *NANOG* were both normalized following the ΔΔCt-method (applying qbase^PLUS^ software), against the geometric average of two different reference sets: on the one hand for *ACTB*, *GAPDH* and *PPIA* (blue triangle); on the other hand for *B2M*, *RPL13A* and Alu repeats (green rhomb). The resulting values represent the fold change in expression levels of the pluripotency factors, and are displayed for *POU5F1* and *NANOG* for both normalization methods in respectively panel A and B. Data shown are obtained from differentiation Experiment 2 (sample isolation every 24 h during 6 days of differentiation).

As can be expected, this comparison revealed a substantial difference in the change of expression levels of *POU5F1* and *NANOG*, emphasizing the importance of proper reference gene selection. As illustrated for Experiment 2 in Figure 2, the decrease in expression of these pluripotency factors is significantly less pronounced using the ‘traditional’ reference genes (*GAPDH*, *ACTB* and *PPIA*) than with the most stable reference loci as defined in this study (*B2M*, *RPL13A* and AluSq) (ANOVA, p-value = 1.30e-05). Also for the other two experiments a significant difference was seen (p-value = 0.0034 and = 0.0215 for Experiment 1 and 3 respectively).

## Discussion

Human ES cells provide a unique opportunity to study early development and may hold great potential for regenerative medicine [3,5,8,11]. The transcription factors Oct4 (*POU5F1*) and Nanog (*NANOG*) contribute to self-renewal and are required for maintaining the pluripotent state of hES cells [6,9]. Therefore, the expression of these factors is commonly assessed with RT-qPCR, as mRNA levels decrease significantly within a few days after inducing differentiation [10,11]. Morphological evaluation of the differentiating cells shows that hES cells start to accumulate at the edges of the colonies and that individual cells adopt a more lengthened, neuronal-like phenotype during early hES cell differentiation (Figure 1) [25].

Adequate RT-qPCR normalization is essential for valid data interpretation. However, in vitro hES cell differentiation entails massive gene expression alterations in general and specifically due to the differentiation agent itself, whereby the expression of individual reference genes may vary, thus making it difficult to select the most suitable and stable references [10,15]. Synnergren and colleagues (2007) already made note of a unique reference gene expression pattern when differentiating hES cells spontaneously [20]. And as Willems et al. (2006) also showed, normalization results vary significantly depending on the reference used [19].

In this study, the suitability of 12 different references was evaluated using the geNorm^PLUS^ algorithm [14,21]. From our results, it can be concluded that *B2M*, *RPL13A* and Alu repeats (AluSq) are the most stable reference loci for this specific differentiation protocol. The use of two references was shown to be sufficient for accurate normalization of RT-qPCR data, though generally the use of three stable references is recommended in literature [14].

The importance of selecting the most stable and suitable references is illustrated by normalization of gene expression levels of pluripotency factors Oct4 and Nanog. Comparison of *B2M*, *RPL13A* and AluSq with more ‘traditional’ reference genes, resulted in a significantly different normalization, indicating that classic reference genes such as *GAPDH* are not always appropriate for a given set-up. In the field of stem cell differentiation, optimization is required for each specific differentiation protocol.

Despite the comparability of different algorithms for determining reference stability (Normfinder, Bestkeeper, Comparative Delta-Ct method), their application may result in a slightly different stability order in comparison with the geNorm^PLUS^ applet. This may indicate though that different reference sets are applicable, resulting in analogous normalization data. When applying those algorithms to the results of Experiment 1, Alu repeats and *RPL13A* remain among the Top 3 stable reference loci (data not shown). *B2M* deteriorates a few ranks when using Normfinder and Bestkeeper, and is replaced by *TBP* as a more stable reference. Nevertheless, the comparison of normalization data for *B2M*, *RPL13A* & AluSq and *TBP*, *RPL13A* & AluSq, gave no significant difference (p-value < 0.5). In conclusion, *B2M*, *RPL13A* and AluSq are suitable reference loci for this experimental set-up of retinoic acid induced hES cell differentiation.

*β-2-microglobulin* (*B2M*) is a component of major histocompatibility complex I, hence being expressed in every nucleated cell, and has yet been applied before as a normalization scalar in different set-ups [26-28]. *Ribosomal protein L13A* (*RPL13A*) is involved in the process of transcript translation, and has also been widely included as a reference gene for RT-qPCR analyses [29-32], in spite of possible presence of pseudogenes [33]. However, pseudogene detection does not necessarily imply that a specific reference is not usable. The main point of interest is the reference stability, which in this case is clearly maintained as supported by the data described above. In our hands both *B2M* and *RPL13A* repeatedly do come forward as a stable reference, supporting their use as normalization genes.

The fact that Alu repeats were one of the best scoring reference loci in the described analyses is not surprising. Because of their genome-wide distribution, they can be considered as a measure for the total amount of mRNA, and the overall Alu element expression will not be influenced by a variation in expression of individual genes [16,22,34]. For this reference, primer specificity is of minor importance. The more sequences are detected by the assay, the less the impact of individual expression variations on the total Alu content. Hence, Alu repeats provide a new strategy for reliable normalization of RT-qPCR data, in particular in experiments where dramatic changes are expected. An additional advantage when working with limited amounts of starting material, is that the input can be lowered, since Alu repeats are highly expressed and thus lead to low Cq values [16].

## Conclusions

This study shows that some of the commonly used reference genes cannot always be included as a stable normalization scalar. Selection of suitable references is highly dependent on the experimental set-up, as is illustrated here for early hES cell differentiation induced by retinoic acid. Furthermore, a new normalization strategy based on Alu repeat expression is proposed and validated for hES cell (retinoic acid induced) differentiation experiments.

## Methods

### Human embryonic stem cell cultures and sample preparation

Human ES cells (UGENT 1 and UGENT2 cell line) were generated in-house [35]. The cells were cultured in 6-well dishes or flasks on a nearly confluent layer of Mitomycin C (Sigma-Aldrich, Steinheim, Germany, #M4287) treated mouse embryonic fibroblasts (MEF, passage 3), in Knock-out DMEM (#41965-039) supplemented with KO-serum replacement (#10828-010), antibiotics (PenStrep, #15140-122), L-glutamine (#25030-024), basic fibroblast growth factor (bFGF, #13256-059), non-essential amino acids (#11140-035, all culture medium products purchased from Invitrogen, Carlsbad, CA, USA) and beta-mercaptoethanol (Sigma-Aldrich, #M7522) and incubated at 37°C, 5% CO_2_.

Differentiation of hES cells was induced by adding 2 μM retinoic acid (Certa, Braine-l’Alleud, Belgium, #640327 T) to the culture medium, and eliminating bFGF. Cells were harvested using 0.25% trypsin-EDTA (Invitrogen, #15596-026) and glass beads (Sigma-Aldrich, #Z265926-1EA). For Experiment 1 hES cells (passage 30) were isolated every 24 hours during 8 days after onset of differentiation, plus an extra sample on day 12. In Experiment 2 (passage 43), samples were also collected every 24 hours, during 6 days. For Experiment 3 (passage 32), samples were collected every 4 hours during day 3, 4 and 5 after differentiation was induced. For each time point, approximately 2 × 10^5^ cells were isolated.

The hES cells were split two days before the start of a differentiation experiment, using 1% collagenase (Type IV, Invitrogen, #17104-019) and glass beads.

### Microscopy

Phase contrast and bright field images of the hES cell culture were acquired with an Axiovert 25 light microscope (Carl Zeiss, Munich, Germany) (objective magnification 5×) and a Sony Alpha 100 camera.

### RNA isolation and RNA quality assessment

After isolation, the cells were immediately resuspended in 1 mL of TRIzol (Invitrogen, #15596-026) and stored at −80°C. For RNA isolation, 200 μL chloroform (Sigma-Aldrich, #C2432) was added to the thawed samples, with subsequent phase separation and purification using an RNeasy Mini kit (Qiagen, Valencia, CA, USA, #74104). After DNase treatment (Qiagen, #79254) and a washing step, RNA was eluted and concentrated using Vivacon 500 spin columns (Sartorius Stedim Biotech, Aubagne Cedex, France, #VN01H32).

RNA quality was assessed for a representative set of samples (6 extra samples taken during Experiment 1) by means of microfluidic capillary electrophoresis. An RNA HighSens Chip (Experion, Bio-Rad Laboratories, Hercules, CA, USA, #7007105) was used to determine a 18S/28S rRNA ratio and an RNA quality index (RQI), after determining RNA concentrations using a Quant-iT RiboGreen RNA kit (Invitrogen, #R11490). The 18S/28S rRNA ratio’s ranged from 1.59 to 1.78 and the RQI’s from 8.7 to 9.9, which indicate good quality samples.

### cDNA synthesis

Complementary DNA (cDNA) was synthesized using a Superscript II kit with oligo(dT) primers (Invitrogen, #11904-018). The cDNA concentration was determined with a Quan-iT OliGreen ssDNA Assay kit (Invitrogen, #O11492), using a spectrophotometer (Tecan, Männedorf, Switzerland). Samples were stored afterwards at −20°C.

### Reverse transcription quantitative PCR

Two different devices were used for RT-qPCR. When using the LightCycler 480 (Roche, Basel, Switzerland), for each reaction 2 μl of cDNA (2.5 ng/μl) was mixed with 3 μl of mastermix, in a 384-well plate. Utilising the ABI Prism 7000 Sequence Detection System (Applied Biosystems, Foster City, CA, USA), each reaction consisted 5 μl of cDNA (2 ng/μl) and 20 μl of mastermix, in a 96-well plate. The same thermocycling conditions were applied for both systems: 2 min at 95°C before 45 cycles of 15 sec at 95°C followed by 1 min at 60°C. When applicable, an additional heating step from 60°C to 95°C was added to obtain melting curves.

The mastermix comprises the primers, iTaq Supermix with ROX (Bio-Rad Laboratories, #1725855) and water. Depending on the locus of interest, probes were included or iTaq Supermix containing SYBR Green (#1725851) was used.

The primers for *ACTB* (Forward: AGAAAATCTGGCACCACACC; Reverse: TAGCACAGCCTGGATAGCAA, SYBR Green detection), and primers and 6-FAM-probes for *PPIA* (F: CAAATGCTGGACCCAATACAAA; R: GCCATCCAACCCCTCAGTCT; Probe: TGTTCCCAGTGTTTCATCTGCACTGCC) and *GAPDH* (F: AGCCTCAAGATCAGCAATG; R: ATGGACTGTGGTCATGAGTCCTT; Probe: CCAACTGCTTAGCACCCCTGGCC) were designed and validated in-house (obtained from Applied Biosystems). These primers were applied at a concentration of 300 nM. The primer sequences for the remaining references (final concentration 250 nM, all detected with SYBR Green) are available in the RTPrimerDB database (http://www.rtprimerdb.org) [36]: *B2M* (RTPrimerDB ID #2), *HMBS* (#4), *HPRT1* (#5), *RPL13A* (#6), *SDHA* (#7), *UBC* (#8), *YWHAZ* (#9). The primer sequences for *TBP* are described in [32]. The sequence of the Alu repeats primers is CATGGTGAAACCCCGTCTCTA for the forward primer and GCCTCAGCCTCCCGAGTAG for the reverse primer. TaqMan assays (Applied Biosystems) were used for the analysis of *POU5F1* (Hs01895061_u1) and *NANOG* (Hs02387400_g1). All reactions were performed in duplo and no template controls were included for all genes. All primer efficiencies lie within the range of 90% to 110%.

### Data analysis

Stability analysis of the different references was performed using the geNorm^PLUS^ application in the qbase^PLUS^ software version 2.0 (Biogazelle) [14,21]. Relative quantification of the pluripotency markers data (Oct4 and Nanog) was calculated using the qbase^PLUS^ software version 2.0. Each sample is relative to a calibrator, in this case undifferentiated human embryonic stem cells (day 0), and is normalized for three reference loci; for *GAPDH*, *ACTB* and *PPIA* on the one hand, or *B2M*, AluSq and *RPL13A* on the other hand.

The relative quantification data for both normalization strategies were statistically analyzed performing an analysis of variance (ANOVA) in R (version 2.13.1).

## Competing interests

The authors declare that they have no competing interests.

## Authors’ contributions

LV conducted the experiments and drafted the manuscript. TO, BH and PDS provided the human embryonic stem cell cultures and commented on the manuscript. CVN performed the statistical analyses. JV participated in the experimental design and commented on the manuscript. DD participated in the experimental design and gave assistance in drafting the manuscript. All authors read and approved the final manuscript.

## Supplementary Material

Additional file 1 **Reference stability analysis: M values.** Reference stability ranking with specific M values, as determined by the geNorm^PLUS^ algorithm, for Experiment 1, 2 and 3. Click here for file
